# Post-Exercise Hypotension and Reduced Cardiac Baroreflex after Half-Marathon Run: In Men, but Not in Women

**DOI:** 10.3390/ijerph17176337

**Published:** 2020-08-31

**Authors:** Laurent Mourot, Alessandro Fornasiero, Mark Rakobowchuk, Laurie Isacco, Alfredo Brighenti, Federico Stella, Andrea Zignoli, Barbara Pellegrini, Cantor Tarperi, Federico Schena

**Affiliations:** 1 EA3920 Prognostic Factors and Regulatory Factors of Cardiac and Vascular Pathologies, Exercise Performance Health Innovation (EPHI) Platform, University of Bourgogne Franche-Comté, 25000 Besançon, France; laurie.isacco@univ-fcomte.fr (L.I.); alfredo.brighenti@gmail.com (A.B.); 2 Division for Physical Education, National Research Tomsk Polytechnic University, 634040 Tomsk, Russia; 3 CeRiSM, Sport Mountain and Health Research Centre, University of Verona, 38068 Rovereto, Italy; alessandro.fornasiero@gmail.com (A.F.); federico.stella91@gmail.com (F.S.); andrea.zignoli@yahoo.it (A.Z.); barbara.pellegrini@univr.it (B.P.); federico.schena@univr.it (F.S.); 4 Department of Neurosciences, Biomedicine and Movement Sciences, University of Verona, 37131 Verona, Italy; cantor.tarperi@univr.it; 5 Department of Biological Sciences, Thompson Rivers University, Kamloops, BC V2C 0C8, Canada; mrakobowchuk@tru.ca; 6 Department of Industrial Engineering, University of Trento, 38123 Trento, Italy; 7 Department of Clinical and Biological Sciences, University of Turin, 10124 Turin, Italy

**Keywords:** baroreflex, sympathetic, parasympathetic, squat stand test, half-marathon, sex, running

## Abstract

We examined whether trained women exhibit similar cardiovascular and cardiac baroreflex alterations after a half-marathon compared to men. Thirteen women (39.1 ± 9.3 years; 165 ± 6 cm; 58.2 ± 7.5 kg; maximal aerobic speed (MAS): 13.7 ± 2.2 km·h^−1^) and 12 men (45.7 ± 10.5 years; 178 ± 7 cm; 75.0 ± 8.3 kg; MAS: 15.8 ± 2.2 km·h^−1^) ran an official half-marathon. Before and 60-min after, cardiovascular variables, parasympathetic (heart rate variability analysis) modulation and cardiac baroreflex function (transfer function and sequence analyses) were assessed during supine rest and a squat-stand test. Running performance was slower in women than in men (120 ± 19 vs. 104 ± 14 min for women and men, respectively). However, when expressed as a percentage of MAS, it was similar (78.1 ± 4.6% and 78.2 ± 5.4% of MAS for women and men, respectively). Before the run, women exhibited lower mean blood pressure (BP), cardiac output (CO) and stroke volume (SV) compared to men, together with higher parasympathetic indexes. After the race, parasympathetic indexes decreased in both sexes, but remained higher in women. Reduced SV, systolic BP and cardiac baroreflex were observed in men but not in women. Contrary to men, a competitive half-marathon did not trigger post-exercise hypotension and a reduced cardiac baroreflex in women.

## 1. Introduction

Cardiovascular disease (CVD) is the leading cause of mortality amongst women worldwide [1,2], making the reduction of CVD risk a crucial factor in reducing mortality [3]. A healthy lifestyle that reduces the risk of CVD should include at least 150 min per week of moderate-intensity aerobic exercise, 75 min per week of vigorous-intensity aerobic exercise or an equivalent combination of the two intensities [4,5].

Accordingly, women are more and more involved in leisure-time running and competitive running events, including running races from 5 km to ultramarathons in distance (>42.2 km) [6,7]. Despite the female sex being well represented in all the different race distances, recent surveys about running event participation reveal that women make up a greater proportion of participants in shorter distance events when compared to longer events [8]. For instance, at races in Switzerland, the number of females completing half marathon is ~12 times higher than marathons [9].

Long-duration and intense physical challenges may reveal cardiac dysfunction that is otherwise compensated for at rest, and a U-shaped relationship between exercise and cardiac morbidity exists [5,10]. Fortunately, the overall risk of sudden death during exercise is considered low (between 0.1 and 38/100,000 person-years), and comparable to that of the general population, meaning that 20% of all sudden death cases are still recorded during exercise [11]. Most deaths can be attributable to underlying cardiac abnormalities where exercise is a mere trigger for a fatal event rather than the actual cause of death [11], together with changes in the autonomic nervous system (ANS) activity.

Indeed, dynamic exercise is associated with a shift towards sympathetic dominance during the exercise and after its cessation [12,13,14], potentially leading to an increase in susceptibility to sudden cardiovascular events [15]. In particular, post-exercise recovery (mainly in the first 30 min and especially after vigorous exercise [16]) is a critical phase for sudden cardiovascular events. This is attributable to increased sympathetic and decreased parasympathetic nerve activity [17]. Depending on exercise and individual’s characteristics, complete autonomic recovery may take even longer [18]. Whether a specific sex-difference in cardiovascular events triggered by exercise is still debated, since studies both suggest a lower atrial fibrillation risk in women but also an increased risk at lower intensities of exercise [19]. The ANS responses need additional research as well.

Indeed, it is well established that at rest, young, pre-menopausal women have greater parasympathetic activity and reduced sympathetic activity [20,21]. This leads to a different cardiovascular regulation by the ANS with lower resting blood pressure (BP) values in women, and they tend to experience orthostatic hypotension and fainting more frequently than men [22]. Whether these autonomic differences persist during exercise and into early recovery is unclear and seems to depend on the training status and the type of exercise. For instance, greater vagal withdrawal during ramp-type exercise below the anaerobic threshold has been suggested in sedentary women [23]. On the contrary, during an acute supramaximal exercise (Wingate test), a lower sympatho-adrenergic response has been reported in female compared to male athletes [24]. Paradoxically, after such an exercise, a greater parasympathetic withdrawal during the recovery was reported in women [25]. Overall, it suggests that despite the fact that women exhibit a more favorable resting autonomic profile, they experience greater autonomic alterations after a single bout of supramaximal exercise. Alongside these observations, hemodynamic determinants of post-exercise hypotension (post-exercise reduction in BP) are likely to differ between sexes and need further investigation especially with intense exercise involving trained participants [26,27].

However, autonomic and cardiovascular responses to endurance exercise have been poorly studied in trained women, despite years of recognition that sex influences physiological responses to exercise. In recent decades, many research groups have pointed out this weakness [28] and it is essential to further characterize women’s response in this area. Thus, the aim of our study was to investigate the effect of an acute, competitive endurance exercise bout (21 km run competition) on cardiovascular and autonomic responses in trained women and men. In accordance with previous observations made after intense exercise, our hypothesis was that trained women would show greater alterations in cardiovascular and parasympathetic responses to a half-marathon than trained men.

## 2. Materials and Methods

### 2.1. Participants

This cross-sectional study involved 25 volunteer amateur runners: 13 healthy, non-pregnant, pre-menopausal women with regular menstruation (menstrual cycle ranges from 25 to 32 days) and 12 men. Although limited, this sample size is in accordance with previous studies on ANS using a similar design [25,29,30,31]. They were recruited within the Run for Science event, hosted by the University of Verona (Italy) in April 2019 [31]. The inclusion criteria were a history of regular recreational running training (mean training regimen of 220 min/week) for more than five years and having previously finished a half-marathon in the previous two years. The presence of disease, pharmacological treatment, cigarette smoking, alcohol (more than six glasses per week) or coffee (more than four cups per day) abuse were exclusion criteria determined by standard medical examination. All participants provided their written informed consent before participating in the experiments. The study was approved by the local Ethical Committee (Department of Neurosciences, Biomedicine and Movement Sciences, University of Verona, Verona, Italy; protocol number 165038) and performed in accordance with the Helsinki Declaration of 1975.

### 2.2. Study Protocol

Maximal oxygen uptake ($\dot{V}$O_2max_), maximal aerobic speed (MAS) and the speeds associated with the first (VT1) and second (VT2) ventilatory thresholds were determined by an incremental treadmill running test at the laboratory, following procedures already described [32]. VT1 and VT2 were determined with the “respiratory equivalent” method, based on breath by breath measures of $\dot{V}$O_2_, carbon dioxide production ($\dot{V}$CO_2_) and ventilation ($\dot{V}$E), with the values being averaged every 10 s. The $\dot{V}$E/$\dot{V}$O_2_ and $\dot{V}$E/$\dot{V}$CO_2_ ratios were plotted against time during the incremental exercise test. VT1 corresponds to a first nonlinear increase in the $\dot{V}$E/$\dot{V}$O_2_ curve, while the $\dot{V}$E/$\dot{V}$CO_2_ slope remains constant. In addition, VT2 is indicated by the nonlinear increase in the $\dot{V}$E/$\dot{V}$CO_2_ curve concomitant to a second strong increase in $\dot{V}$E/$\dot{V}$O_2_ with a further increase in exercise intensity. Briefly, the protocol test was individualized for each participant to control the duration of each test (incremental phases lasted 8–12 min). Therefore, the initial speed was determined by the participant’s capacity, and it was increased by 0.5 km/h every minute until exhaustion. The running surface slope was kept at a constant +1% throughout the test (Runrace Technogym, Gambettola, Italy). Oxygen uptake and ventilatory parameters were determined breath-by-breath using a Cosmed metabolic cart (Quark PFT, Cosmed Rome, Italy).

No more than 15 days later, participants competed in an official half-marathon race certified by the Italian Track and Field Federation. The day of the race, the weather was sunny, with no wind, the air temperature was 19 °C with 71% humidity (stable throughout the duration of the event). Participants were instructed to fast for at least 3 h before testing, to refrain from ingesting beverages containing caffeine and alcohol and not to exercise (beyond normal lifestyle activities) for at least 24 h prior to testing. To avoid many people reaching the testing station simultaneously, the participants started the race in waves (from 7:30 to 10:00 a.m.) scheduled based on their individual estimated race time. Before, and 1 h after the cessation of the exercise, participants in underwear were weighed to the nearest 0.1 kg with a digital scale (Seca, Hamburg, Germany). Cardiovascular variables, including heart rate (HR), systolic (SAP) and diastolic (DAP) arterial blood pressures, were then measured continuously (Portapres^®^; Finapres Medical System, Amsterdam, The Netherlands) over a 10-min period while the participants lay in the supine position. Additionally, R-R intervals were measured continuously using a Polar RS800CX HR monitor (Polar, Kempele, Finland). Resting data were used to obtain spontaneous changes in arterial blood pressure, R-R interval and baseline steady-state hemodynamics. Cardiovascular parameters were also collected during repeated squat-stand maneuvers (Squat Stand Test, SST) performed for 5 min with a duty cycle of a squat held for 10 s followed by 10 s standing [31]. During SST, the participants were instructed to avoid performing a Valsalva maneuver while standing up.

### 2.3. Heart Rate Variability, Baroreflex Sensitivity and Hemodynamic Assessment

The Portapres^®^ device measures arterial pressure using photoplethysmography of the middle phalanx of the middle finger, which is calibrated to the oscillometrically obtained brachial BP. Arterial pulse pressure (PP, mmHg) was calculated by subtracting DAP from SAP. The HR/Inter-Beat Interval (IBI) was derived from the beat-to-beat arterial pressure wave.

The arterial pressure signal was then analyzed using Beatscope Software (TNO-TPD, Biomedical Instrumentation) to estimate other cardiovascular variables. Stroke volume (SV) was estimated using the Modelflow method [33,34], and cardiac output (CO) was calculated as the product of HR and SV, whilst total peripheral resistance (TPR) was determined by dividing the mean arterial BP (MAP) by the CO. Additionally, arterial pressure was measured in the right arm by an electro-sphygmomanometer (Omron Healthcare, Kyoto, Japan) to corroborate the BP measurements from the Portapres^®^ device. IBI and SAP values extracted from Portapres^®^ device were used for subsequent baroreflex sensitivity (BRS) analysis [31].

### 2.4. Data Analysis

Mean values of BP (SAP, DAP and MAP), other hemodynamic variables (SV, CO and TPR) and BRS and HRV indexes were calculated from the last 5 min of the 10-min period during supine rest and from the entire 5 min of the squat stand test (SST).

Beat-by-beat SAP and IBI values were used to assess cardiac baroreflex sensitivity (BRS). SAP and IBI data were linearly interpolated and resampled at 2 Hz for spectral and transfer function analysis (TF). Under resting conditions, TF of gain, phase and coherence between spontaneous oscillations in SAP and IBI were calculated in accordance with the work of Zhang et al. [35], i.e., 0.05–0.15 Hz for the low frequency (LF) range. During SST (performed at 0.05 Hz) TF gain, phase and coherence were calculated across a specific frequency (SF) range (i.e., 0.031–0.078 Hz). Cardiac BRS was also assessed with the sequence method [36]. The sequence method is based on the identification of at least three consecutive beats (sequence) in which a defined increase (or decrease) in SAP is followed by a defined increase (or decrease) in the IBI. Only sequences with a minimum correlation coefficient of 0.85 were accepted. Positive and negative sequences were averaged to obtain a representative value of cardiac baroreflex sensitivity (BRS_seq_). To better represent BP control in the increasing and decreasing directions, mean gain values of positive (BRS_Seq_+) and negative (BRS_Seq_−) sequences were also computed separately.

As described and independently, R–R intervals obtained using the Polar RS800CX heart rate monitor were uploaded to the Polar Precision Performance software (Polar, Kempele, Finland) and then exported as text files. HRV analysis was performed using Kubios HRV software (Version 2.1, Biosignal Analysis and Medical Imaging Group, Kuopio, Finland [37]). Signal artifacts were filtered by means of a moderate error correction filter. All the time series of R–R intervals showed low noise (identified errors <5%). As the physiological significance of several HRV indexes is still disputed [38], only indexes of parasympathetic modulation were calculated in the time domain (square root of the sum of successive differences between adjacent normal R–R intervals squared; RMSSD) and in the frequency-domain high-frequency spectral power (HF, 0.15–0.4 Hz), calculated by Fast Fourier Transform (FFT) [39]. The respiratory rate was neither controlled nor recorded. However, on an individual basis, we systematically checked that the respiratory sinus arrythmia peak fell within the HF band. All recordings were consistent in this regard.

### 2.5. Statistical Analysis

Data are presented as mean ± SD. The normal distribution of the data was verified with the Shapiro–Wilk test. If data were not normally distributed, natural logarithm transformation (Ln) was applied to obtain a normal distribution and to allow parametric statistical comparisons. All the variables were normally distributed after this procedure. Two-tailed unpaired t-tests were used to compare running times during the run between the two groups. A two-way (time (pre vs. post) × group (men vs. women)) repeated measures analysis of variance (ANOVA) followed by Holm–Sidak post hoc analyses was performed to assess the effects of run and group on all other variables. A *p*-value of <0.05 was considered statistically significant.

## 3. Results

The height (*p* < 0.001) and weight (*p* < 0.001) of women (164.8 ± 6.1 cm and 58.2 ± 7.5 kg, respectively) were significantly reduced compared to men (178.3 ± 6.7 cm and 75.0 ± 8.3 kg, respectively). Both women and men were normal-weight according to their body mass index values, the values being, nevertheless, significantly higher in women than in men (21.4 ± 1.8 vs. 23.6 ± 2.3 kg·m^−2^ for women and men, respectively, *p* = 0.012). No significant difference in age (*p* = 0.110) was observed (39.1 ± 9.3 and 45.7 ± 10.5 years for women and men, respectively). The $\dot{V}$O_2_max (*p* = 0.018) and MAS (*p* = 0.034) of women (46.8 ± 6.8 mL·kg^−1^·min^−1^ and 13.7 ± 2.2 km·h^−1^, respectively) were significantly lower than for men (51.3 ± 8.5 mL·kg^−1^·min^−1^ and 15.8 ± 2.2 km·h^−1^, respectively). The speeds associated with VT1 (*p* = 0.049) and VT2 (*p* = 0.041) were significantly lower for women (10.6 ± 1.5 and 11.8 ± 1.8 km·h^−1^, respectively) than for men (11.7 ± 1.2 and 13.4 ± 1.5 km·h^−1^, respectively).

**Figure 1:**
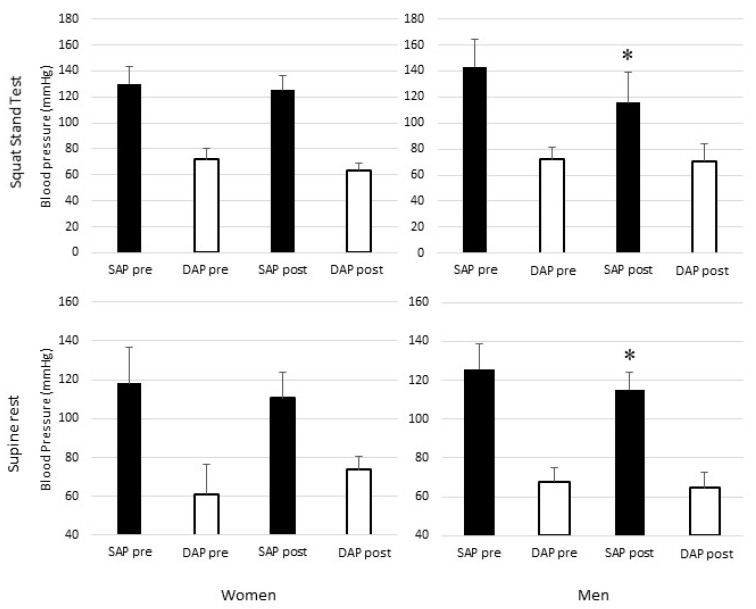
Mean ± SD Systolic (SAP; black bars) and diastolic (DAP; white bars) blood pressures before (Pre) and after (Post) the 21-km run in men (**left** panels) and women (**right** panels) in supine position (**up** panels) and during the squat stand test (**bottom** panels). SAP = systolic arterial blood pressure, DAP = diastolic arterial blood pressure; pre = before the half-marathon run; post = after the half-marathon run; * = significantly different from pre.

Hemodynamic variables and indexes of ANS function during supine rest are presented in Figure 1 (bottom) and Table 1. Before the run, women exhibited a significantly lower SAP, MAP, CO and SV compared to men, whilst other hemodynamic variables were not different (i.e., HR and TPR). They also exhibited significantly higher Ln-RMSSD, Ln-HF and HFnu, as well as significantly lower coherence-LF from transfer function analysis.

Hemodynamic variables and indexes of ANS during SST are presented in Figure 1 (top) and Table 2. Before the run, no hemodynamic and autonomic variables were different between women and men, except a significantly lower SV and CO in women.

Absolute running performance was significantly slower amongst women than men (half-marathon time (*p* = 0.028) and mean speed (*p* = 0.024) were 120.1 ± 19 min and 10.8 ± 1.6 km·h^−1^ and 104.4 ± 13.7 min and 12.3 ± 1.5 km·h^−1^ for women and men, respectively). However, when expressed as percentage of MAS, the speed of their performances was very similar (78.1 ± 4.6 and 78.2 ± 5.4% for women and men, respectively, *p* = 0.962). This represented 90 ± 6% and 92 ± 6% of the speed associated with VT2 (*p* = 0.412) and 101 ± 7% and 105 ± 8% of the speed associated with VT1 (*p* = 0.199) for women and men, respectively, without significant differences between the two groups.

No significant differences between groups were observed for both absolute (*p* = 0.102) and relative weight loss (*p* = 0.376) after the half-marathon (−0.94 ± 0.58 kg (−1.51 ± 0.61%) and −1.13 ± 0.67 kg (−1.62 ± 0.78%) and for women and men, respectively).

During supine rest after the 21-km race, HR was significantly increased in both groups (Table 1). SAP, MAP and SV significantly decreased in men only. Ln-RMSSD significantly decreased in both groups, with the values for women remaining significantly higher than for men. Ln-HF significantly decreased in men only, with Ln-HF being significantly lower than in women. In women only, HFnu significantly decreased. The number of positive and negative sequences significantly increased in men only, with the values being significantly higher than in women. On the contrary, during supine rest after the run, the baroreflex sensitivity assessed by the sequence method was significantly higher in women than in men. Additionally, cardiac BRS assessed by the transfer function method showed a significant decreased in men only (gain-LF).

During SST after the race, the mean HR significantly increased in both groups (Table 2). SAP, SV and CO decreased in men only. Ln-RMSSD and Ln-HF significantly decreased in both groups. Gain BRS-SF significantly decreased in men only. Phase BRS-SF significantly increased in women only.

## 4. Discussion

The aim of the present study was to compare the cardiovascular and autonomic changes triggered by a competitive 21-km run (half-marathon) in women and men. We hypothesized that women would show greater autonomic and cardiovascular alterations, but the results did not confirm our hypothesis. The main finding was that women did not exhibit significant post-exercise hypotension and cardiac BRS impairment during supine rest or during a dynamic task challenging the BP control via orthostatic stress (5-min squat stand test), maintaining a significantly higher parasympathetic nervous activity than men after a competitive half-marathon.

In our study, women were smaller and lighter than men. Accordingly, they exhibited smaller SV and CO at rest before the half-marathon (Table 1) [21]. We also observed a lower MAP in women. This is in accordance with repeated observations suggesting that women have a lower prevalence of hypertension, and tend to have lower BP than men, until the age of menopause [20,22,40,41], when the incidence of these processes accelerate and reach those of men rapidly [22]. The ANS activity may partly explain this observation since women tend to have lower muscle sympathetic nerve activity [42]. Also, higher indexes of cardiac vagal control have been reported in both young and middle-age women [21,25,29,43], as in the present study. Hence, our results are in accordance with the observation that women typically display greater parasympathetic control of HR than men at rest [20,21]. Several studies have reported that compared to men, resting cardiac BRS is decreased in young women [20,44,45,46], but this finding is not universal, as other studies have also reported no differences [20,30,47,48], such as is the present study (Table 1). While cardiac BRS is linked to parasympathetic activity, increased HRV indexes do not necessarily means increased cardiac BRS, as already observed [49]. Taken together, these data are in line with the view that men and women display different autonomic and cardiovascular profiles at rest, with the latter showing higher parasympathetic activity and lower BP levels.

In addition to the parasympathetically mediated difference, men and women differ in terms of anthropometric and body composition characteristics as well as hormonal status, which confers physiological advantages upon men in sport performance [50]. However, the sex dimorphism in performance depends on the sport discipline and competition duration. Men may benefit from their larger body size, muscle mass and greater strength, and maximal anaerobic and aerobic capacity particularly during sports involving high power output. Conversely, the sex difference in performance is smallest in long distance disciplines, as observed with ultramarathon run for example [30]. While in the present study the same relative speed (≈78% of MAS) was observed in both groups, men completed the 21-km faster than women due to their higher MAS. For both groups, the observed performance was in the range of expected values for half-marathon participants of similar training levels (e.g., [51]). The longer exercise duration for women resulted in a similar weight loss (that could be used as a rough estimation of hydration status), which was in the range of typical weight loss observed with a half-marathon race [31].

As expected in response to the race, the two groups showed a significant increase in HR together with a significant reduction in HRV parasympathetic-derived indexes, both at rest and during SST (Table 2). Despite this reduction, parasympathetic indexes remained significantly higher in women than in men (Table 2), suggesting that even after strenuous exercise, healthy pre-menopausal trained women still exhibit cardioprotective autonomic profiles [52].

On the other hand, significantly decreased SV, SAP and MAP (i.e., post-exercise hypotension) were observed in men only, highlighting dissimilar post-exercise cardiovascular responses between sexes. The selective decrease in SV has been reported after 60 min of cycling at 60% of peak oxygen uptake [27], but not after longer endurance-type exercise such as an ultramarathon [30]. Together with methodological considerations (i.e., posture, time after exercise, method used to assess SV), this discrepancy is likely due to the relative intensity maintained during the exercise (the lower the longer the exercise), and to the possibility (or not) of water ad libitum. As well, a significant decrease in SAP and MAP at rest (i.e., post-exercise hypotension [53]) was observed in men only, and was accompanied by a decrease in cardiac BRS (both transfer function and sequence method) and an increase in the number of positive and negative sequences to compensate for the reduced BRS slope. Interestingly, during the dynamic task (SST), significant systolic blood pressure reduction was observed despite absence of significant reduction in BRS. It implies that the mechanisms triggering post-exercise hypotension are different during resting and dynamic conditions. Unfortunately, the present data do not allow us to give a satisfactory explanation of this phenomenon. Further studies are thus required to specifically address the question of a different post-exercise regulation at rest and under dynamic conditions.

Cardiac BRS is important in regulating BP, and post-exercise alterations depend upon the intensity of the prior exercise. For instance, 30 min of exercise at 65% of HRmax leads to cardiac BRS improvement [54], whilst high-intensity (>85% HRmax) and maximal aerobic exercises (conducted to exhaustion) may result in decreased post-exercise cardiac BRS, which generally recovers within 60 min of exercise termination [55,56]. In the range of intensities used in our study, likely above >85% HRmax and at an average intensity of 78% of MAS and 90% of the speed associated to the second ventilatory threshold maintained for 100–120 min, cardiac BRS responses may be more variable due to dual autonomic control of the heart or thermal influences on HR [53]. Unfortunately, only men were involved in these previous studies, and for instance, Senitko et al. [27] did not evaluate cardiac BRS in their comparison of sedentary and trained women and men after 1 h of cycling. No sex differences in cardiac BRS have been reported after ultra-endurance exercise (135 ± 50 km, over 28 ± 9 h) [30] but in that case, the nature of the exercise stimuli is very different from the present study (beyond the important duration, the intensity for such race is low (e.g., [57])). Hence, to date, sex differences in post-exercise cardiac BRS responses have been poorly studied and do not specifically relate to endurance exercise [49,58], making comparison with the existing literature difficult. Due to the scarce evidence available, the combination of exercise intensity and duration that can trigger a significant sex influence on post-exercise BRS requires further study and clarification. Yet our work suggests, for the first time, dissimilar post-exercise cardiac BRS responses between men and women.

The limitations of the present study relate to its design. The design of this study is cross-sectional, and we cannot exclude that factors other than sex were responsible for our observations, such as different characteristics linked to training history. Also, the specific organization of this study did not allow us to recruit more participants and limited the sample size [59]. Despite this limit, most of the observed significant differences were associated with the appropriate statistical power of 80%. Previously, work that reported sex differences following laboratory-based investigations used similar sample sizes (e.g., [25,30]), and we do not believe this factor impacted our results, although we cannot exclude the possibility of a type II error. However, confirmation of our results with a larger sample size is mandatory.

Also, this study limits our ability to obtain detailed information about women’s menstrual cycle phases and contraceptive habits. It is known that exercise recovery depends on menstrual cycle phase, at least within muscle [60]. However, previous work suggests that cycle phase does not affect BRS as well as the gain around the operating point in young women [20]. Hence, it is possible that cardiovascular and ANS evaluations in the present study may have been different in the women in other phases of their menstrual cycle. However, this would have introduced more variability into the data, and since we were able to demonstrate significant group differences in key outcomes, we do not believe this impacted our results. Age also matters in SAP control, since the higher vagal activity seen in young women tends to disappear with increasing age [22]. The similar baroreflex responses between men and women observed after ultratrail running [30] were amongst 45 years-old women, an age group slightly older than ours (39 years-old). Beyond this age-difference, the history of exercise training needs to be better quantified as well. Indeed, different training characteristics could lead to different ANS [61] and blood pressure [62] responses. Also, this study may have benefitted from a better method of evaluating the race characteristics, including the evaluation of pacing strategy (i.e., positive or negative split), race load (using, e.g., Edward’s TRIMP [63]), cardiac strain (using, e.g., the Physioflow device [64]) and hydration status, to assess any potential differences in hemodynamic variables. Finally, a difference of up to 19% (0.3 L·min^−1^) has been reported when comparing Modelflow to thermodilution [65], and thus, an alternative method could have been used, at least to calibrate the Modelflow. Because of theses limits and although we did measure some mechanistically relevant variables like catecholamines, we have not focused on the mechanisms and further studies are needed to confirm our observations and examine any underlying mechanisms.

## 5. Conclusions

Overall, our results showed that a competitive 21-km run did not trigger post-exercise hypotension during supine rest or during a dynamic task designed to challenge BP control mechanisms via orthostatic stress (5-min repeated squat stand test) in women. Despite a significant reduction post half-marathon, women were able to maintain a significantly higher parasympathetic nervous activity compared to men, with a preserved cardiac baroreflex sensitivity. Our results support the idea that aerobically trained healthy premenopausal women are able to cope with the stresses of competitive running and continue to display a cardioprotective autonomic profile after strenuous aerobic exercises. Further research investigating the sex differences in post-exercise autonomic and cardiovascular responses (with special regard to the hemodynamic determinants of post-exercise hypotension and baroreflex responses to various exercises) is needed.

## Figures and Tables

**Table 1:** Hemodynamic and neuro-hormonal variables before (Pre) and after (Post) the 21-km run in women and men in supine position.

REST																
		Women							Men							
		Pre			Post				Pre				Post			
Hemodynamics																
HR	bpm	60.2	±	10.5	68.3	±	10.9	*	60.4	±	7.8		74.4	±	9.0	*
SV	mL	78	±	21	75	±	21		97	±	18	#	75	±	20	*
CO	L/min	4.7	±	1.6	5.1	±	1.8		5.9	±	0.9	#	5.7	±	1.7	
SAP	mmHg	118	±	12	110	±	6		126	±	13	#	115	±	9	*
DAP	mmHg	61	±	13	59	±	7		68	±	7		65	±	8	
MAP	mmHg	79	±	12	76	±	5		87	±	6	#	81	±	9	*
TPR	mmHg per L·min^−1^	1.19	±	0.51	1.06	±	0.54		0.90	±	0.17		0.91	±	0.42	
Heart Rate Variability																
IBI	ms	1029	±	179	904	±	152	*	1010	±	127		819	±	103	*
Ln-RMSSD	ms	3.94	±	0.64	3.61	±	0.64	*	3.43	±	0.34	#	2.91	±	0.34	*,#
Ln-HF	ms^2^	6.51	±	1.14	5.99	±	1.53	*	5.61	±	0.78	#	4.40	±	0.94	*,#
HFnu		52	±	14	41	±	14	*	27	±	12	#	24	±	13	*,#
Transfer Function Analysis																
Gain-VLF	ms mmHg^−1^	5.1	±	1.5	6.0	±	2.9		4.7	±	1.5		4.8	±	1.7	
Gain-LF	ms mmHg^−1^	8.7	±	6.1	9.8	±	8.8		8.1	±	2.7		5.7	±	2.5	*
Gain-HF	ms mmHg^−1^	2.43	±	0.49	2.35	±	0.85		2.11	±	0.26		1.77	±	0.46	#
Phase-VLF	rads	0.10	±	1.10	0.03	±	0.71		−0.17	±	0.67		0.06	±	0.71	
Phase-LF	rads	−0.59	±	0.38	−0.49	±	0.56		−0.75	±	0.28		−0.61	±	0.25	
Phase-HF	rads	0.08	±	0.48	−0.06	±	0.36		−0.16	±	0.27		−0.31	±	0.19	#
Coh-VLF		0.47	±	0.08	0.53	±	0.16		0.47	±	0.10		0.53	±	0.05	
Coh-LF		0.44	±	0.16	0.46	±	0.17		0.58	±	0.09	#	0.54	±	0.15	
Coh-HF		0.40	±	0.18	0.44	±	0.17		0.46	±	0.13		0.45	±	0.15	
Sequence Method																
n seq+		4	±	2	4	±	3		5	±	3		8	±	5	*,#
n seq−		6	±	3	4	±	2		6	±	3		9	±	5	*,#
BRS-seq+	ms mmHg^−1^	12.6	±	10.1	16.3	±	10.1		11.2	±	5.7		8.3	±	3.6	#
BRS-seq−	ms mmHg^−1^	13.7	±	10.6	19.1	±	15.4		11.7	±	7.9		9.2	±	5.5	#
BRS-seq	ms mmHg^−1^	13.3	±	9.6	16.5	±	10.1		11.5	±	4.7		9.2	±	4.7	#

HR = Heart beat; SV = Stroke Volume; CO = Cardiac Output; SAP = systolic arterial blood pressure; DAP = diastolic arterial blood pressure; MAP = Mean arterial blood pressure; TPR = total peripheral resistance; IBI = inter-beat-interval; RMSSD = square root of the sum of successive differences between adjacent normal R–R intervals squared; HF = High Frequency; nu = normalized units; VLF = Very Low Frequency; LF = Low Frequency; Coh = Coherence; n seq = number of sequences; BRS = Baroreflex sensitivity; pre = before the half-marathon run; post = after the half-marathon run; * = significantly different from PRE; # = different from women; *p* < 0.05.

**Table 2:** Hemodynamic and baroreflex function variables before (Pre) and after (Post) the 21-km run in women and men during squat stand test.

Squat Stand Test																
		Women							Men							
		Pre			Post				Pre				Post			
Hemodynamics																
HR	bpm	84	±	10	95	±	11	*	81	±	11		101	±	8	*
SV	mL	61	±	23	66	±	16		96	±	18	#	58	±	20	*
CO	L/min	5.1	±	2.2	6.0	±	1.4		7.6	±	1.8	#	5.6	±	2.1	*
SAP	mmHg	130	±	16	125	±	23		143	±	28		116	±	17	*
DAP	mmHg	72	±	14	64	±	21		72	±	13		71	±	18	
MAP	mmHg	91	±	14	84	±	19		95	±	17		85	±	19	
TPR	mmHg per L.min^−1^	1.18	±	0.63	1.10	±	0.52		0.84	±	0.24		0.93	±	0.45	
Heart Rate Variability																
IBI	ms	737	±	87	655	±	85	*	766	±	97		605	±	50	*
Ln-RMSSD	ms	3.92	±	0.56	3.53	±	0.51	*	3.80	±	0.39		3.14	±	0.33	*,#
Ln-HF	ms^2^	6.47	±	1.10	5.45	±	1.11		6.22	±	1.06	*	4.96	±	0.78	*
HFnu		9	±	8	5	±	3		9	±	13		6	±	3	
Transfer Function Analysis																
Gain-SF	ms mmHg^−1^	3.72	±	2.43	3.76	±	2.49		3.82	±	1.98		2.36	±	1.00	*
Phase-SF	rads	−0.69	±	0.39	−0.97	±	0.32	*	−0.93	±	0.39		−0.96	±	0.37	
Coh-SF		0.62	±	0.20	0.64	±	0.10		0.70	±	0.10		0.72	±	0.11	
Sequence Method																
n seq+		12	±	2	13	±	3		11	±	6		9	±	5	
n seq−		15	±	3	14	±	3		13	±	4		14	±	3	
BRS-seq+	ms mmHg^−1^	7.03	±	5.11	8.09	±	7.58		4.79	±	1.24		5.11	±	2.80	
BRS-seq−	ms mmHg^−1^	6.13	±	4.32	5.74	±	3.65		3.61	±	1.12		4.25	±	2.15	
BRS-seq	ms mmHg^−1^	6.65	±	4.31	6.83	±	5.34		4.00	±	1.09		4.66	±	2.04	

HR = Heart beat; SV = Stroke Volume; CO = Cardiac Output; SAP = systolic arterial blood pressure; DAP = diastolic arterial blood pressure; MAP = Mean arterial blood pressure; TPR = total peripheral resistance; IBI = inter-beat-interval; RMSSD = square root of the sum of successive differences between adjacent normal R–R intervals squared; HF = High Frequency; nu = normalized units; VLF = Very Low Frequency; LF = Low Frequency; Coh = Coherence; n seq = number of sequences; BRS = Baroreflex sensitivity; * = significantly different from PRE; # = different from women; *p* < 0.05.
